# A molecular barcode to inform the geographical origin and transmission dynamics of Plasmodium vivax malaria

**DOI:** 10.1371/journal.pgen.1008576

**Published:** 2020-02-13

**Authors:** Ernest Diez Benavente, Monica Campos, Jody Phelan, Debbie Nolder, Jamille G. Dombrowski, Claudio R. F. Marinho, Kanlaya Sriprawat, Aimee R. Taylor, James Watson, Cally Roper, Francois Nosten, Colin J. Sutherland, Susana Campino, Taane G. Clark

**Affiliations:** 1 Faculty of Infectious & Tropical Diseases, London School of Hygiene & Tropical Medicine, London, United Kingdom; 2 Department of Parasitology, Institute of Biomedical Sciences, University of São Paulo, São Paulo, Brazil; 3 Shoklo Malaria Research Unit, Mahidol-Oxford Tropical Medicine Research Unit, Faculty of Tropical Medicine, Mahidol University, Mae Sot, Tak, Thailand; 4 Harvard T.H. Chan School of Public Health, Department of Epidemiology, Boston, Massachusetts, United States of America; 5 Broad Institute of MIT and Harvard, Cambridge, Massachusetts, United States of America; 6 Centre for Tropical Medicine and Global Health, Nuffield Department of Clinical Medicine Research Building, University of Oxford Old Road Campus, Oxford, United Kingdom; 7 Mahidol Oxford Research Unit, Faculty of Tropical Medicine, Mahidol University, Bangkok, Thailand; 8 Faculty of Epidemiology and Population Health, London School of Hygiene and Tropical Medicine, London, United Kingdom; University of Pennsylvania, UNITED STATES

## Abstract

Although *Plasmodium vivax* parasites are the predominant cause of malaria outside of sub-Saharan Africa, they not always prioritised by elimination programmes. *P*. *vivax* is resilient and poses challenges through its ability to re-emerge from dormancy in the human liver. With observed growing drug-resistance and the increasing reports of life-threatening infections, new tools to inform elimination efforts are needed. In order to halt transmission, we need to better understand the dynamics of transmission, the movement of parasites, and the reservoirs of infection in order to design targeted interventions. The use of molecular genetics and epidemiology for tracking and studying malaria parasite populations has been applied successfully in *P*. *falciparum* species and here we sought to develop a molecular genetic tool for *P*. *vivax*. By assembling the largest set of *P*. *vivax* whole genome sequences (n = 433) spanning 17 countries, and applying a machine learning approach, we created a 71 SNP barcode with high predictive ability to identify geographic origin (91.4%). Further, due to the inclusion of markers for within population variability, the barcode may also distinguish local transmission networks. By using *P*. *vivax* data from a low-transmission setting in Malaysia, we demonstrate the potential ability to infer outbreak events. By characterising the barcoding SNP genotypes in *P*. *vivax* DNA sourced from UK travellers (n = 132) to ten malaria endemic countries predominantly not used in the barcode construction, we correctly predicted the geographic region of infection origin. Overall, the 71 SNP barcode outperforms previously published genotyping methods and when rolled-out within new portable platforms, is likely to be an invaluable tool for informing targeted interventions towards elimination of this resilient human malaria.

## Author summary

*Plasmodium vivax* is the most widespread parasite causing human malaria, with more than one-third of the world’s population being at risk of infection. *P*. *vivax* is resilient due to its dormant liver phase, and there are increasing reports of drug-resistance and life-threatening infections. Despite this, *P*. *vivax* malaria is not always prioritised by elimination programmes. New molecular tools are needed to inform elimination efforts, including through better understanding the geographical source and outbreaks of *P*. *vivax*, thereby leading to the halting of transmission and the targeting of reservoirs of infection. Our work describes a 71 genetic marker barcode for *P*. *vivax* that has high predictive ability to identify the geographic origin, and has the potential to distinguish local transmission networks. If the 71 genetic marker barcode is implemented within new portable molecular platforms, it is likely to be an invaluable tool for informing targeted interventions towards elimination of this resilient human malaria.

## Introduction

*Plasmodium vivax* is the predominant cause of malaria outside of sub-Saharan Africa [1,2] and there are increasing reports of drug-resistance and severe complications that pose a threat to children and pregnant women [3–7]. Elimination efforts have led to reductions in the prevalence of the deadlier *P*. *falciparum* malaria, but areas of co-endemicity have seen a corresponding rise in the proportion of *P*. *vivax* infections, which appear more resilient to control strategies [8]. In order to halt transmission of vivax, malaria programmes should focus on identifying the main reservoirs of infection and target control measures towards these. *P*. *vivax* has been observed in regions where malaria transmission had once been interrupted [9], and in such a context, continuing surveillance and the use of genetic tools to identify transmission networks within malaria outbreaks is essential. The utility of genetic tools has been demonstrated in previous studies in low-transmission settings, such as in Malaysian Borneo [10] or places where there are imported cases, such as in Greece [11]. Further, understanding the transmission dynamics of the parasite populations through assessment of genetic diversity has the potential to play a key role in guiding the elimination efforts, including by revealing transmission events and identifying potential foci of infection. In recent years, genomic studies have dissected the molecular dynamics of *P*. *vivax* populations in regions with stable transmission [12–16]. Other studies have used microsatellites to study trends in the population dynamics [17–20]. The availability of whole genome sequencing data can inform the design of “genetic barcodes” which require low numbers of SNP polymorphisms [21] but can be used to infer transmission networks and the geographic source of infections. Molecular barcodes, when combined with affordable delivery systems, can facilitate *P*. *vivax* epidemiological and surveillance investigations.

Microsatellite markers have been used to reveal a spectrum of population structures in *P*. *falciparum* [22], as well as infer transmission dynamics and complexity of infections [23]. Compared to microsatellites, SNP markers are more suitable for comparisons of both strongly and weakly diverged populations, and in revealing ancestral patterns of genetic structuring [24]. By leveraging off whole genome sequencing for population characterisation [14,15,25,26], a number of SNP-based barcodes have been derived for *P*. *falciparum*. A barcode based on 24 SNPs in the nuclear genome (“24-SNP barcode”) has been used to identify and track isolates from an endemic population in Senegal [27]. This barcode harboured capacity to identify clones from non-clones [21] using data from a limited set of long-term adapted laboratory lines [27], and has been employed on field isolates to infer local temporal changes in genetic diversity [28]. This approach has demonstrated the potential effectiveness of such tools in combination with epidemiological methods to elucidate transmission intensity in malaria endemic regions [28]. Nevertheless, the use of isolates with a very limited geographical spread to generate the barcode can underestimate the genomic variability present in other *Plasmodium* populations and lead to low precision when estimating relatedness [21], thereby impeding the transportability of the 24-SNP barcode across global malaria regions. It has been shown that the predictive power of the 24-SNP barcode for geographical determination is poor, especially when compared to one formed of 23 SNPs from the mitochondria and apicoplast organellar genomes, which predicted the continental origin of samples with 92% accuracy [29]. Another barcode formed of 105 highly frequent nuclear genome SNPs was developed to infer transmission intensity using a geographically broader panel of isolates, thereby potentially providing greater utility across malaria-endemic countries [30]. Simulations on genome-wide data from *P*. *falciparum* has recommended the use of at least 200 barcoding SNPs for identity by decent (IBD) analysis in haploid eukaryotes [21]. Overall, the studies in *P*. *falciparum* have demonstrated that SNP barcodes can potentially provide insights into the intensity of transmission, identify the geographical origin of the field isolates, and inform the dynamics of the diversity in a parasite population. These include outbreak identification, an event which has been shown to be more likely in low-transmission settings [31].

SNP barcodes for *P*. *vivax* have been proposed, including one based on 42-SNP nuclear polymorphisms and another on mitochondrial genome markers, but both developed to ascertain the source of infection [32,33]. One limitation of these barcodes is that they were based on relatively small datasets. As more data becomes available and geographical coverage increases, genotyping tools with greater predictive power and wider global reach can be developed [14–16,34]. Technological advancements in genomics can be leveraged, including the high throughout sequencing of candidate genomic regions and portable genotyping [35]. Ultimately, the identification and integration of informative loci for *P*. *vivax* and other plasmodium parasites for inferring transmission and infection source has the potential to revolutionise global malaria surveillance. Here, using whole genome sequencing data for 433 *P*. *vivax* isolates across 17 countries, we applied machine learning and SNP tagging approaches from human genome-wide association studies (GWAS) to create a 71 SNP barcode with high predictive ability for geographic origin (91.4% accuracy) and the capability to infer transmission. We demonstrate that the barcode outperforms alternative approaches, including microsatellite genotyping, for global geographical profiling and inferring outbreak events within a low-transmission setting in Malaysia. Further, we validate the barcode by analysing the 71 SNPs in *P*. *vivax* DNA sourced from 132 recent UK travellers to East Africa, Asia and South America. Our work demonstrates that the 71 SNP barcode has the potential to be an invaluable tool to help elimination efforts of this resilient neglected *Plasmodium* species.

## Results

### SNPs, samples and population structure

We aligned raw sequence data from 867 isolates [12,14–16,34,36] to the PvP01_v1 (http://genedb.org) reference genome, and identified 1,522,046 SNPs. Isolates with high levels of missing genotype calls (> 30%) and high multiplicity of Infection (> 20% heterozygous genotypes) were excluded from analysis. The final dataset was formed of 433 isolates (S1 Table), spanning 17 countries and 6 regions (East Africa 25; South Asia 4; Southeast Asia 242; Papua New Guinea 26; South America 116; North America 20), and 720,340 high quality SNPs of which 89.7% were non-common (minor allele frequency (MAF) < 5%) (S1 Fig, **left**), consistent with previous findings [34].

**Fig 1:**
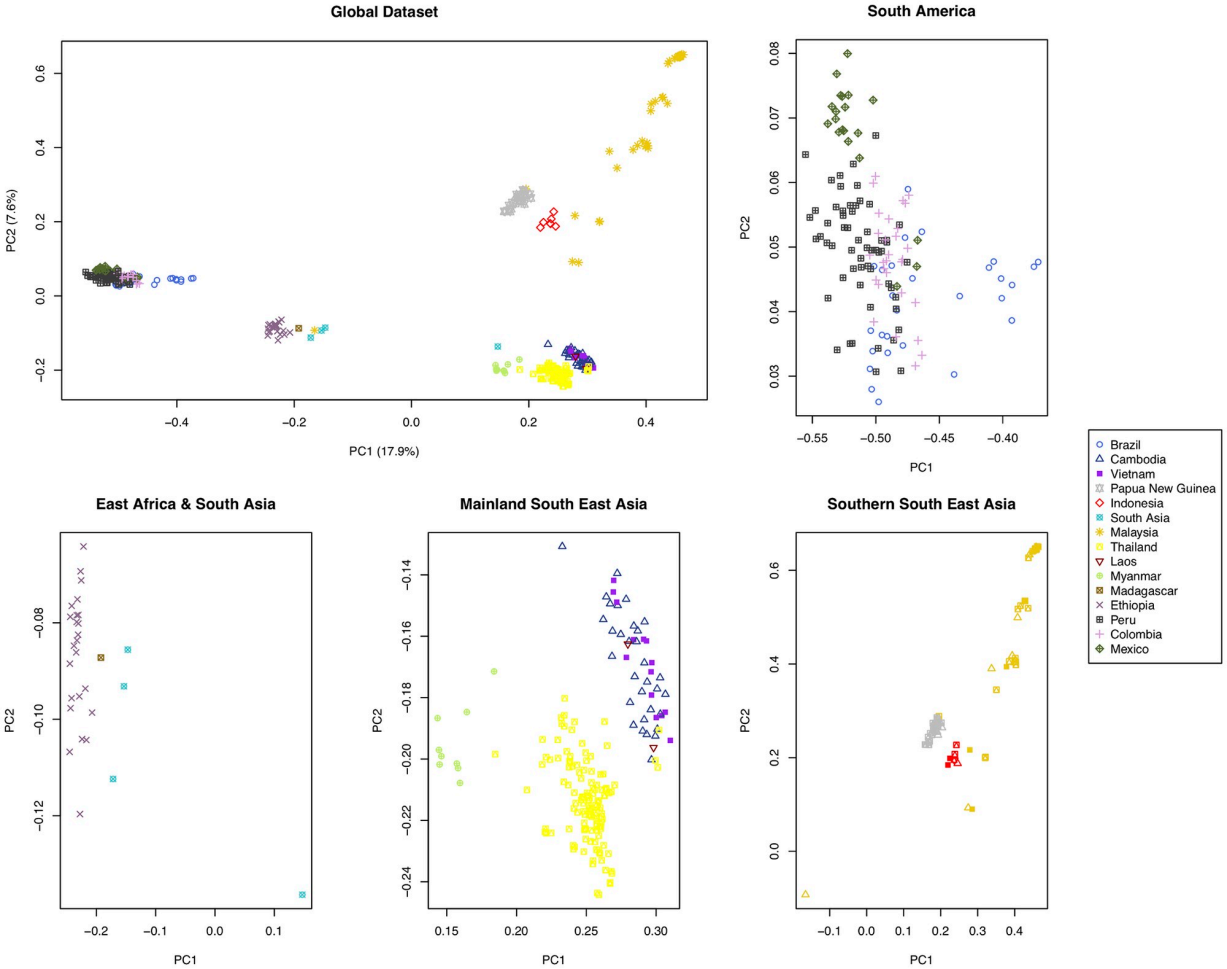
Principal component (PC) analysis plot generated using 720,340 high quality SNPs across 433 *P*. *vivax* isolates reveals geographic clustering. Isolates are coloured according to country of origin. Clustering by region can be observed, with Southeast Asian isolates appearing to group at the bottom right of the plot, Oceania at the top right, and South American isolates on the centre left. A relative degree of clustering by country can be observed, especially for isolates from Oceania and to a lesser extent Southeast Asia. The percentage of variation explained for each PC is shown in the axis labels. Additional region-specific plots are shown for clarity.

**Fig 2:**
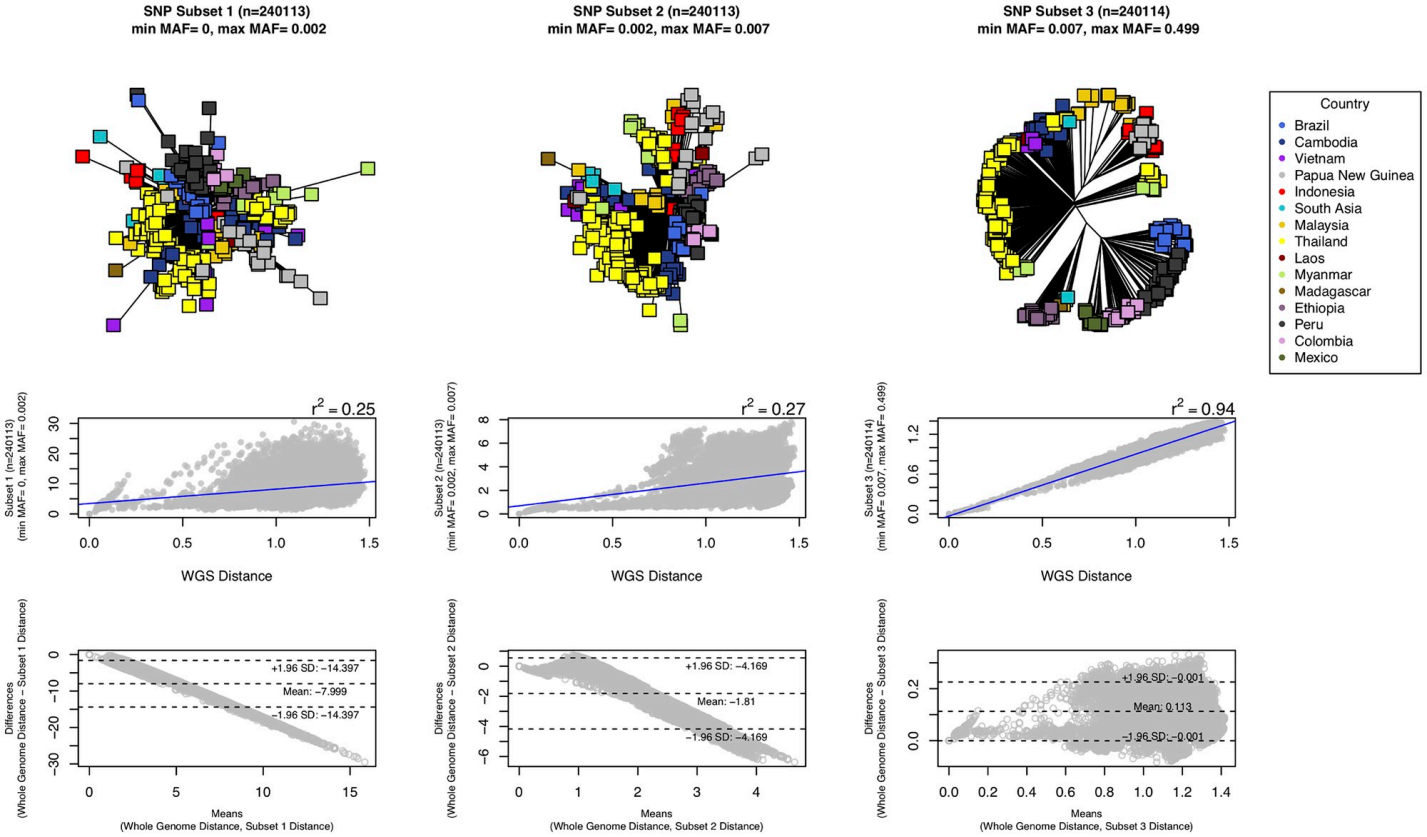
The sub-setting of SNPs by minimum allele frequency (MAF) reveals the strong explanatory power of high frequency SNPs in *Plasmodium vivax*. Three equally sized groups of SNPs were constructed from the distribution of the minor allele frequencies: (**Left**) [MAF 0–0.2%], (**Centre**) [MAF 0.2–0.7%] and (**Right**) [MAF 0.7–50%]. **(Top)** Each of these subsets was used to construct a neighbour-Joining tree revealing only a clear geographic clustering in the high frequency SNP group [MAF 0.7–50%]; **(Middle)** The Pearson’s *r*^*2*^ correlation of the genome distance calculation using all SNPs and each subset separately, reveals the poor correlation for the low frequency SNPs (*r*^*2*^: [MAF 0–0.2%; left] 0.25, [MAF 0.2–0.7%; middle] 0.27) and a strong correlation for the high frequency subset ([MAF 0.7–50%; right] *r*^*2*^ = 0.94); **(Bottom)** A Bland-Altman analysis comparing the differences in genetic distance between using whole genome SNPs (“gold standard”) and each of the SNP subsets. This reveals that subsets of SNPs with low MAF tend to overestimate the distance (panels left and centre, with mean differences -7.99 and -1.81 respectively; SD = standard deviation). Whilst, in the high MAF subset (right) the genetic distance was underestimated (mean of differences: 0.113).

A principal component analysis (PCA) revealed that the isolates clustered broadly by regional groups: Southeast Asia (Thailand, Myanmar, Cambodia, Vietnam and Laos), South Asian/East Asian/Southeast Asia (China), Americas (Peru, Colombia, Mexico and Brazil) and Oceanian/Southeast Asia (Papua New Guinea, Indonesia and Malaysia) and others located around the Arabian Sea (Fig 1). The genomic distance between intra-country isolates was on average smaller (mean: 26,505 SNPs, range: 0–37,801 SNPs) compared to between inter-border isolates (mean: 33,160 SNPs, range: 6,034–39,676 SNPs), in line with previous studies [14,15]. The notable exception to the pattern were isolates sourced from neighbouring Thailand and Myanmar, supporting evidence they belong to a similar parasite population [14], which matches with the Thai samples being collected in the western region [15]. Despite this exceptional situation, our analysis suggests the potential of identifying markers that could determine geographical origin to a country level in most settings. Intuitively, the highly skewed distribution of the MAF towards rare variants suggests that more common SNPs are those driving the population structure observed. To assess this hypothesis, we split the dataset into three equally sized divisions based on SNP MAF (tertiles: < 0.002, 0.002–0.007, > 0.007; S1 Fig, **right**), and for each we constructed neighbour-joining trees and correlated the pair-wise genome distance with the estimate based on all 720k SNPs (Fig 2). These comparisons revealed the strong explanatory power harboured by those SNPs with the highest MAF (Pearson’s *r*^*2*^ correlation of 0.94 with the full 720k SNP set) compared to the subsets with lower MAF (Pearson’s *r*^*2*^ correlations of 0.25 and 0.27). A MAF cut-off (> 0.3) was determined (S1 Fig, **right**), leading to a subset of 16,110 SNPs used as the starting point for barcode building for country classification.

### Selection of highly informative barcoding SNPs using a tagging and machine learning approach

In order to further reduce number of markers used for barcoding construction, we applied the software *TAGster* [37] to identify tagging SNPs that summarise blocks of high linkage disequilibrium (LD), as estimated by the *r*^*2*^ metric across windows of size 500 kbp. The genetic variability can be captured by tagging SNPs due to the strong LD structure found in *P*. *vivax* populations [34], and we identified 1,173 SNPs, which summarised variation in 4,896 neighbouring SNPs, almost 40% of the total dataset. The 1,173 highly informative SNPs were then used as the pool for a further selection using a random forest modelling approach. Prior to implementation, missing alleles (4.2%) were imputed with high accuracy (< 1% error, see Materials and methods), and a neighbour joining tree was constructed using the resulting data, thereby confirming that no bias was introduced to the clustering patterns (S2 Fig, **top**). The correlation between genome distances based on the 1,173 SNPs and whole genome sequencing (720k SNPs) was high (Pearson’s *r*^*2*^ = 0.96) (S2 Fig, **middle**). Similarly, a Bland-Altman analysis revealed the genetic distances between isolates obtained using the subset of 1,173 tagging SNPs were similar to those based on whole genome SNPs (S2 Fig, **bottom**), especially those with the highest MAF (see Fig 2, **bottom right**). We sought to show that the 1,173 SNPs could not only predict differences between geographical regions, but also estimate genetic distances within intra-border isolates and identify subclades in low transmission settings [31]. In particular, when analysing those isolates within a genomic distance equivalent of <20,000 SNPs (0.74), we found a high correlation (Pearson’s *r*^*2*^ = 0.98) between distances based on the 1,173 tagging and 720k genome-wide SNPs. This analysis revealed that the 1,173 high frequency tagging SNPs can not only detect strong inter-border differentiation but potentially identify highly related isolates within the same country based on genomic distance.

Application of the random forest classification approach to the classification of country involved constructing 500 trees, partitioning the dataset randomly into 80% training (n = 346) and 20% for validation (n = 87), and using 34 variables at each nodal split. These default settings have been used previously in *P*. *falciparum* genome-wide analysis with success [38]. Classification error rates became stable when 100 trees were averaged (S3 Fig, **B**). The final model inside the training set performed with an overall out-of-bag error rate of 17.1%, where the main classification errors were found across Southeast Asian populations (Thailand, Vietnam, Cambodia, and Myanmar). These populations were identified as being highly related using the SNP-based genomic distance (Fig 2, **right**), supporting previous observations [14]. The random forest model was then used to identify the 60 SNPs with the highest predictive importance across the trees. The 60 SNP cut-off was established using a point of inflection analysis of cumulative predictive importance, where the addition of further SNPs does not significantly improve predictive power (S3 Fig, **B**). A further 11 SNPs were chosen to summarise high between-country genetic differentiation based on the fixation index (*F*_*ST*_ > 0.7), leading to a final barcoding set of 71 SNPs (Table 1). Within this SNP set, there are differences in allele frequency across the two main regions (Southeast Asia and South America), but no markers were fixed across the populations. The 71 markers are in low linkage disequilibrium (S4 Fig) (LD *r*^*2*^: mean 0.15, inter-quartile range 0.02–0.24), but some blocks of correlation are observed, which can be explained by an uneven geographic distribution of the isolates.

**Table 1:** Selection of 71 barcoding SNPs for *Plasmodium vivax*. The barcode has the ability to predict geographic origin and perform transmission inference. Using SNPs without complete fixation, clustering is observed across populations, and there is an increased number of haplotypes that can be traced and therefore assist the potential identification of transmission events. Countries/regions are included if they have at least two samples.

Chr*	Position	Alleles**	Gene***	Peru	Colombia	Mexico	Brazil	Ethiopia	Cambodia	Vietnam	Thailand	Laos	Myanmar	South Asia	PNG****	Indonesia	Malaysia
6	531114	A/G	0612100	1.00	1.00	1.00	1.00	0.92	0.00	0.00	0.00	0.00	0.00	0.50	0.00	0.00	0.00
11	1548740	G/T	.	1.00	1.00	0.00	0.82	0.00	0.00	0.00	0.00	0.00	0.00	0.00	0.00	0.00	0.00
8	1508826	A/G	0835600	0.98	1.00	1.00	1.00	0.04	0.00	0.00	0.00	0.00	0.00	0.00	0.92	0.43	0.02
12	2568422	C/T	.	0.98	1.00	1.00	0.96	0.00	0.00	0.00	0.00	0.00	0.00	0.00	0.00	0.00	0.00
13	924465	T/A	.	0.97	1.00	1.00	0.82	0.46	0.09	0.00	0.00	0.00	0.00	0.25	0.77	0.57	0.08
12	2741883	T/C	1265900	0.95	0.33	0.00	0.79	0.00	0.00	0.00	0.00	0.00	0.00	0.00	0.00	0.00	0.00
8	1364769	A/G	.	0.72	0.00	0.40	0.61	0.00	0.00	0.00	0.00	0.00	0.00	0.00	0.00	0.00	0.00
14	1451245	C/T	.	0.03	0.23	0.00	0.82	0.00	0.00	0.00	0.00	0.00	0.00	0.00	0.00	0.00	0.00
14	2799980	C/T	.	0.79	1.00	1.00	0.89	1.00	0.09	0.00	0.02	0.00	0.00	0.75	0.96	0.43	0.04
11	1790451	G/A	1142200	0.33	0.63	0.75	0.32	0.00	0.16	0.00	0.05	0.50	0.11	0.00	0.96	0.71	0.90
5	1181738	G/A	.	0.14	0.40	0.00	0.00	0.04	0.00	0.00	0.05	0.00	0.00	0.25	0.85	0.00	0.12
12	287842	A/C	1207100	1.00	0.97	0.95	0.93	0.63	0.00	0.00	0.06	0.00	0.00	0.75	0.00	0.00	0.00
8	59546	C/T	0801100	1.00	1.00	1.00	0.93	0.75	0.03	0.00	0.08	0.00	0.11	0.50	0.00	0.00	0.02
14	1338047	T/C	1430700	0.26	0.87	0.65	0.46	0.04	0.00	0.00	0.10	0.00	0.00	0.25	0.85	0.57	0.86
5	1252615	C/A	.	0.59	0.73	0.35	0.68	0.00	0.03	0.29	0.12	0.00	0.00	0.25	0.46	0.71	0.96
13	593388	T/C	1313200	0.97	1.00	1.00	1.00	1.00	0.00	0.00	0.13	0.00	0.44	1.00	0.08	0.00	0.02
9	1728276	G/T	.	0.93	1.00	1.00	0.82	1.00	0.06	0.00	0.13	0.00	0.44	1.00	0.04	0.00	0.02
12	2751196	C/T	.	0.98	0.83	1.00	0.89	0.13	0.03	0.00	0.14	0.00	0.56	0.75	0.00	0.00	0.00
8	115537	A/C	.	0.95	1.00	1.00	1.00	1.00	0.03	0.00	0.15	0.00	0.44	0.50	0.00	0.00	0.72
3	435501	T/A	0309300	0.93	1.00	1.00	0.79	0.67	0.03	0.07	0.15	0.00	0.11	0.00	0.00	0.00	0.00
3	382736	T/C	0307900	0.00	0.13	0.00	0.07	0.67	0.03	0.00	0.15	0.00	0.22	0.75	0.00	0.00	0.04
2	697366	T/G	0216200	0.84	0.77	0.30	0.43	0.71	0.00	0.07	0.16	0.00	0.00	0.75	0.00	0.00	0.02
9	253550	C/A	0903800	0.97	0.90	0.50	0.82	0.00	0.00	0.00	0.17	0.00	0.11	0.25	0.54	0.29	0.02
12	323603	C/T	1208000	0.93	0.87	0.05	0.93	0.08	0.06	0.07	0.18	0.00	0.00	0.25	0.46	0.14	0.92
4	667734	T/G	0416400	1.00	1.00	1.00	0.82	0.92	0.09	0.07	0.24	0.00	0.22	0.75	0.00	0.14	0.04
14	2151758	G/A	.	0.59	0.63	0.15	0.21	1.00	0.16	0.21	0.27	0.00	0.22	1.00	0.88	0.57	0.94
12	2330891	T/C	.	0.95	0.97	1.00	0.96	1.00	0.03	0.00	0.27	0.00	0.56	0.75	0.96	0.86	0.04
14	2096518	A/G	1448200	0.98	1.00	1.00	1.00	1.00	0.19	0.36	0.34	0.50	0.56	0.75	0.88	0.86	0.08
13	815186	A/G	1317300	0.59	0.60	0.30	0.61	0.08	0.03	0.79	0.35	0.00	0.56	0.25	1.00	0.86	0.78
3	101401	T/C	.	0.09	0.80	0.85	0.50	0.75	0.19	0.14	0.44	0.00	0.56	0.50	0.19	0.29	0.34
8	987176	C/A	0822400	0.16	1.00	0.55	0.00	0.08	0.75	0.64	0.46	0.50	0.56	0.00	0.35	0.00	0.14
4	401576	A/G	0409900	0.97	1.00	0.95	1.00	0.00	0.47	0.29	0.47	0.50	0.67	0.75	0.00	0.00	0.02
5	1087255	T/G	0526800	0.93	1.00	1.00	0.68	1.00	0.03	0.00	0.53	0.00	0.89	0.75	0.00	0.00	0.02
8	1546424	C/G	0836700	0.83	0.93	1.00	0.86	0.58	0.16	0.07	0.61	0.00	0.11	0.50	0.00	0.29	0.14
5	606507	A/G	0514500	0.90	0.87	0.25	0.36	0.63	0.19	0.14	0.63	0.00	0.78	0.25	0.00	0.00	0.02
14	1270401	G/C	1429500	0.97	1.00	1.00	0.89	0.96	0.09	0.21	0.66	0.00	0.33	1.00	0.00	0.00	0.02
13	354018	T/C	1307600	0.98	0.97	1.00	1.00	1.00	0.50	0.79	0.70	0.50	0.89	1.00	1.00	0.00	0.10
3	101610	G/A	.	0.71	0.23	0.15	0.14	0.38	0.78	0.57	0.71	1.00	0.56	0.75	0.65	0.57	0.14
3	101866	G/T	.	0.83	0.23	0.15	0.14	0.67	0.69	0.71	0.73	1.00	0.56	1.00	0.85	0.71	0.68
3	101653	A/G	.	0.69	0.10	0.15	0.14	0.46	0.78	0.64	0.73	1.00	0.56	1.00	0.65	0.57	0.68
5	213306	G/A	.	0.10	0.03	0.00	0.04	0.92	0.09	0.00	0.75	0.00	0.67	0.75	0.00	0.00	0.22
9	641801	T/C	0913800	0.02	0.00	0.00	0.00	1.00	0.78	0.57	0.75	0.50	0.67	0.75	0.12	0.57	0.02
12	492536	T/C	.	0.00	0.00	0.00	0.00	0.17	0.84	0.79	0.79	1.00	0.89	0.50	0.00	0.14	0.96
14	1071214	C/T	1424900	0.00	0.00	0.00	0.00	0.00	0.63	0.29	0.80	0.00	0.67	0.00	0.00	0.00	0.76
3	812961	C/G	0319600	0.00	0.00	0.00	0.00	0.00	0.78	0.93	0.83	1.00	0.89	0.25	0.00	0.14	0.90
5	318005	C/T	0507200	0.76	0.03	0.00	0.36	0.92	0.28	0.36	0.86	0.50	0.89	0.75	0.15	0.57	0.96
5	1206169	G/T	.	0.84	0.90	0.60	0.54	0.46	0.47	0.64	0.88	1.00	0.78	0.75	0.77	0.57	0.10
12	629287	T/C	1214900	0.26	0.43	0.95	0.21	0.71	0.84	0.79	0.88	0.50	1.00	1.00	0.04	0.14	0.12
1	157446	T/A	0103400	1.00	0.90	0.10	0.89	0.92	0.50	0.50	0.88	1.00	0.78	0.75	0.27	0.57	0.10
10	1161740	G/C	1026700	0.84	0.90	0.10	0.39	0.79	0.84	1.00	0.88	1.00	0.78	1.00	0.19	0.00	0.04
13	340505	G/A	1307300	0.09	0.17	1.00	0.04	0.17	0.94	0.86	0.88	1.00	0.56	0.75	0.96	0.00	0.94
7	503559	A/G	0709800	0.00	0.00	0.00	0.68	0.00	0.69	0.50	0.88	0.50	0.89	0.75	0.00	0.29	0.52
5	1066232	G/A	0526300	0.00	0.00	0.00	0.00	0.00	0.75	0.86	0.89	1.00	0.78	0.25	0.08	0.14	0.94
3	799618	A/G	0319300	0.28	0.03	0.05	0.25	0.42	0.56	0.57	0.90	1.00	0.78	0.75	0.04	0.14	0.12
12	1534427	T/C	.	0.78	0.80	0.75	0.50	0.08	0.94	0.86	0.91	0.50	0.78	1.00	0.00	0.14	0.04
8	1510168	C/T	.	0.14	0.23	0.05	0.29	0.50	0.69	0.71	0.91	0.50	0.89	0.25	0.73	0.43	0.04
13	1232713	G/A	1329100	0.09	0.00	0.10	0.00	0.00	0.63	0.86	0.91	0.00	0.78	0.50	0.04	0.29	0.02
10	274067	T/C	.	0.00	0.00	0.00	0.00	0.17	0.66	0.64	0.91	1.00	0.67	0.25	0.00	0.29	0.94
11	1372724	C/G	1132000	0.17	0.37	0.45	0.18	0.79	0.50	0.64	0.92	1.00	0.67	1.00	0.35	0.71	0.90
2	155305	G/T	0203000	0.00	0.00	0.00	0.00	0.00	0.97	0.57	0.92	1.00	0.44	0.00	0.00	0.00	0.00
13	1231631	T/C	1329100	0.91	1.00	1.00	0.93	0.08	0.72	0.71	0.93	0.00	0.67	0.75	0.31	0.14	0.10
4	649508	T/A	0415900	0.00	0.00	0.00	0.00	0.17	0.69	1.00	0.93	1.00	0.78	0.00	0.04	0.29	0.36
7	497459	A/G	0709800	0.00	0.00	0.00	0.61	0.00	1.00	0.93	0.94	1.00	1.00	0.25	0.04	0.29	0.98
4	692311	T/C	0416900	0.55	0.63	0.15	0.61	0.75	0.94	0.86	0.95	1.00	0.89	0.75	0.42	0.57	0.14
6	639238	G/A	0615100	0.02	0.80	0.10	0.64	0.63	0.72	0.86	0.95	1.00	0.56	0.50	0.65	0.57	0.94
3	536764	C/T	0312200	0.00	0.00	0.00	0.00	0.17	0.81	0.93	0.95	1.00	0.89	0.00	0.12	0.57	0.96
14	801261	A/T	1418100	0.00	0.00	0.00	0.00	0.00	0.00	0.00	0.95	0.00	0.67	0.25	0.00	0.14	0.92
13	1696786	C/G	.	0.07	0.13	0.00	0.00	0.67	0.88	0.86	0.97	1.00	1.00	1.00	0.04	0.29	0.38
14	585620	C/T	1413400	0.00	0.00	0.00	0.00	0.00	0.94	0.86	0.98	1.00	0.67	0.00	0.00	0.00	0.00
13	769284	C/T	.	0.00	0.00	0.00	0.00	0.04	0.94	1.00	0.99	1.00	1.00	1.00	0.00	0.00	0.94
9	1217464	C/T	0927600	0.00	0.27	0.00	0.36	0.17	1.00	1.00	1.00	1.00	0.89	0.00	0.88	0.71	0.98

* Chromosome;

** Reference Allele / Alternative Allele;

*** PVP01;

**** Papua New Guinea

**Fig 3:**
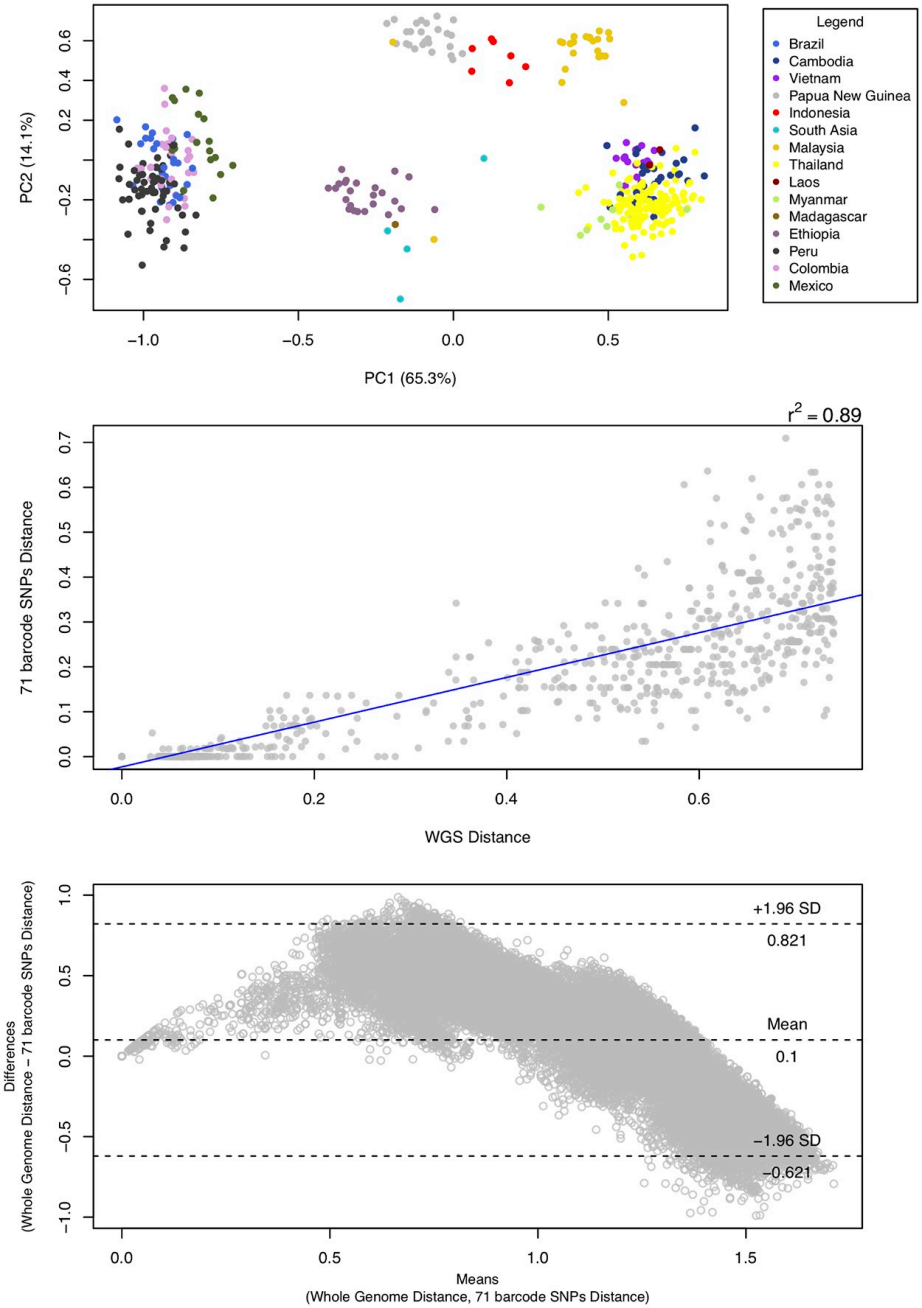
Geographic clustering of *Plasmodium vivax* isolates using the 71 SNP barcode. **(Top)** A principal component (PC) analysis plot shows clustering by region and country when using the 71 SNP barcode. The percentage of variation explained by each PC is shown in the axis labels; **(Middle)** A strong Pearson’s *r*^*2*^ correlation of 0.898 was observed between the genetic distances based on genome-wide (n = 720k) and 71 barcoding SNPs, revealing the potential for the barcode to identify closely related intra-border isolates; (**Bottom**) A Bland-Altman analysis comparing the differences in genetic distance between using whole genome SNPs (“gold standard”) and the 71 SNP barcode.

In order to assess the potential of the barcode for geographic classification, a PCA was generated using only the 71 SNPs (Fig 3, **top**), and similar clustering patterns were observed to those using the genome-wide (720k) SNP set (Fig 1). When comparing the genomic distances obtained using the 71 barcoding to 720k genome-wide SNPs in intra-border pair-wise comparisons, we observed a high Pearson’s *r*^*2*^ correlation (0.898), providing evidence for the potential of this barcode to not only identify geographical origin but also provide insights into the relatedness of intra-border isolates (Fig 3, **middle**). Similarly, a Bland-Altman analysis revealed little overall bias in the estimation of genetic distance between isolates using whole genome and the 71 barcoding SNPs (Fig 3, **bottom**; average difference of 0.09). Using the 71 barcoding SNPs, there is a trend towards an underestimation of the genetic distance for closely related isolates, and an overestimation for distantly related ones. The potential use of the barcode to infer intra-border relatedness and provide insights into transmission dynamics, is supported by the observation that 98.2% (425/433) of the haplotypes obtained were unique in the dataset. Furthermore, the 71 SNPs were used to predict the geographical source of the 20% of the isolates (n = 87) not used to develop the random forest model. The model using only the 71 SNPs yielded an overall accuracy of 91.4% (8.6% out-of-bag error) in predicting the country source of these 87 isolates and a further 16 Brazilian newly sequenced isolates. The 71 SNPs outperformed a published 42-SNP barcode [33], which under the same random forest model conditions (80%/20% training/prediction and 500 trees) obtained a 77.5% accuracy. The low accuracy of the 42-SNP barcode can also be observed in the ambiguous clustering found in the PCA (S5 Fig, **top**) and neighbour-joining tree (S5 Fig, **bottom**). Furthermore, the correlation between genetic distances based on the SNP barcode and genome-wide (720k) SNPs was lower (Pearson’s *r*^*2*^ = 0.59).

### *In-silico* testing of the barcode in a near-elimination setting in Malaysia

**Fig 4:**
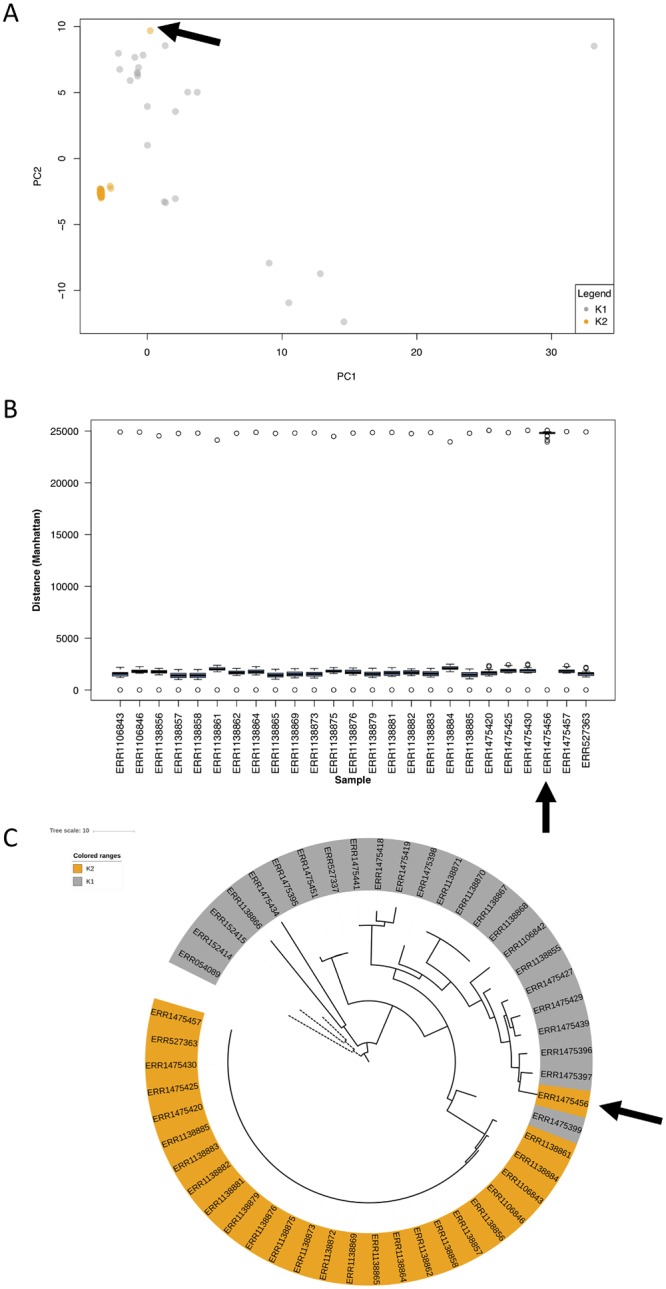
Use of the 71 SNP barcode in *Plasmodium vivax* isolates from Sabah, Malaysia reveals patterns of transmission. A dataset of 60 isolates from a near-elimination setting that has been exhaustively characterised by whole genome sequencing in [31] was analysed here by means of a principal component analysis (PCA) using the 71 SNP barcode from our study. **(A)** The principal component (PC) analysis revealed the previously reported outbreak population (K2, yellow). However, there was one “K2” isolate showing distant clustering (ERR1475456, highlighted with arrows in the three panels); **(B)** The distribution of pairwise genome SNP distances for each of the isolates in the outbreak, showing that ERR1475456 is not as closely related to the outbreak as indicated by microsatellite genotyping in [31]; **(C)** A neighbour-joining tree revealed isolates from the West Coast Division in Sabah (dashed lines) clustering together; isolates are coloured in the tree according to cluster.

A field-ready SNP barcode with the potential for being deployed in low-transmission settings has to be proven efficacious in settings where the identification of foci of infection and imported cases are of key relevance. We used a dataset comprising 60 *P*. *vivax* isolates, sourced from Sabah Malaysia, where the population dynamics have been extensively studied using microsatellite markers and whole genome sequencing [31]. *In silico* characterization using the 71 SNP barcode in a PCA analysis identified two main populations, denoted previously as K1 and K2 (Fig 4A). K2 comprised of 26 almost genetically identical isolates in a known transmission outbreak, previously supported using a set of nine microsatellite markers [31]. The estimated genetic distances between isolates based on the 71 SNPs were strongly correlated with those based on the genome-wide SNPs (Pearson’s *r*^*2*^ = 0.88). The outbreak isolates shared the same haplotype across the barcoding polymorphisms in most cases, with only 3 isolates presenting a one SNP difference. There was one notable exception (ERR1475456) which presented a larger genetic distance (Fig 4A). This isolate shared the same microsatellite haplotype as the outbreak K2, but the genomic distance from the samples in the rest of the outbreak samples was greater, and therefore likely to be of independent origin (Fig 4B). A neighbour-joining tree constructed using the 71 SNPs revealed clustering by administrative division in Sabah Malaysia, indicating its potential for tracking of cases from different health authorities (Fig 4C).

### Prospective testing of the barcode using traveller genotype data

**Fig 5:**
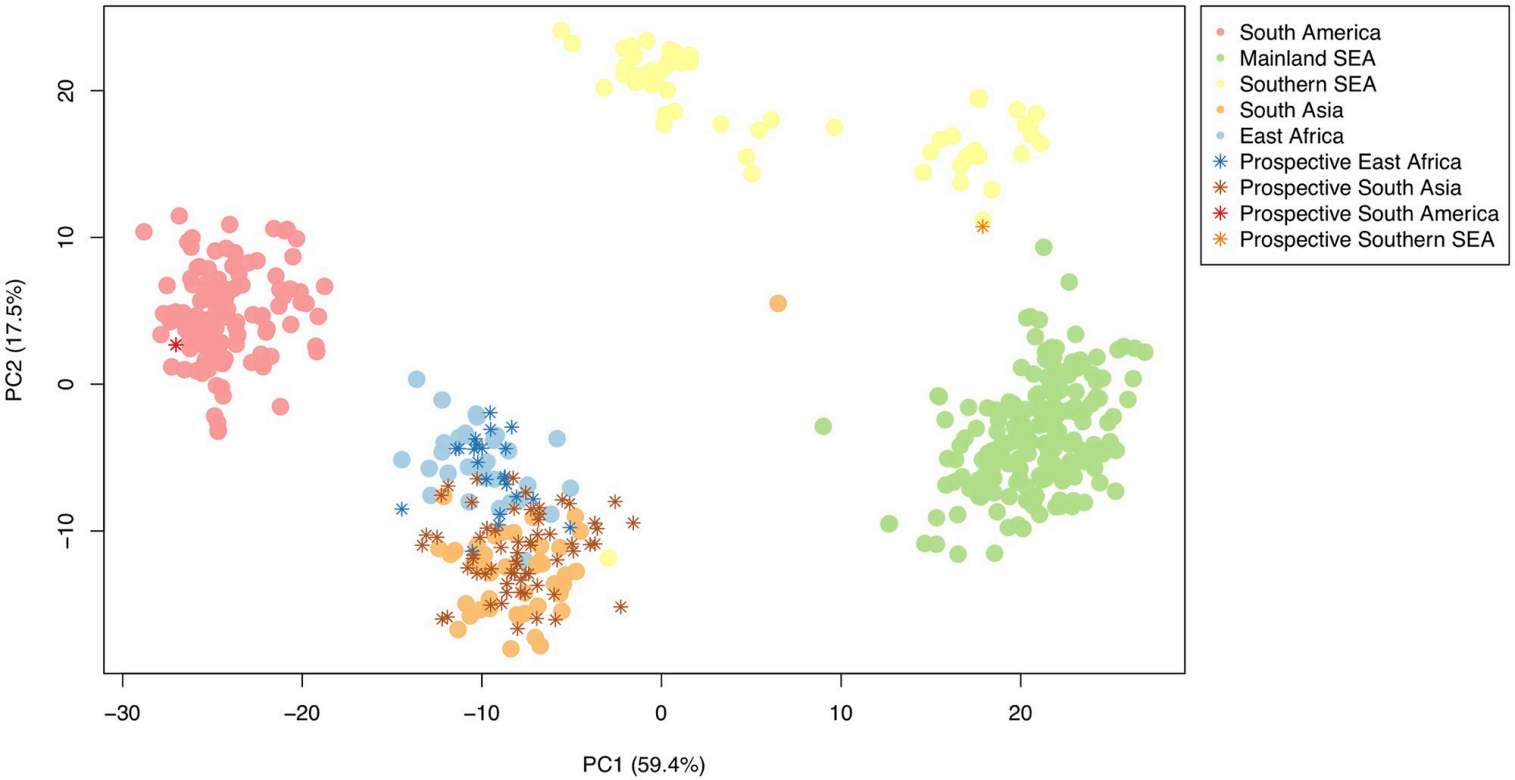
The principal component (PC) analysis plot of the 565 *P*. *vivax* isolates, constructed using the 71 SNP barcode. The isolates include the 433 used in the design of the barcode (circles) and the 132 prospective UK traveller samples (stars). The plot shows clear geographic region clustering, with the traveller samples from each region (strong star-dots for each colour) overlapping with the previously sequenced data (light circular-dots for each colour). The percentage of variation explained for each PC is shown in the axis labels.

The genotypes for the 71 barcoding SNPs were characterised in 132 *P*. *vivax* DNA sourced from returning travellers to the UK from ten endemic countries (n = 132; Afghanistan (n = 26), Bangladesh (n = 1), Eritrea (n = 11), Ethiopia (n = 6), Guyana (n = 3), India (n = 38), Pakistan (n = 35), the Philippines (n = 1), Sudan (n = 7) and Uganda (n = 4)). The genotypes were used to construct a combined PCA plot for the 132 prospective and 433 barcode-development isolates, which revealed clear clustering by geographic region (Fig 5). The isolates co-localised in the PCA with the previously determined wider-regional populations, including East Africa, South Asia, Southern Southeast Asia and South America. A PCA based only on prospectively collected data (n = 132) (S6 Fig) revealed a clear regional pattern, but no strong clustering at a country level. The country source was predicted using the 71 SNP barcoding genotypes for each prospective isolate. An 80.1% country-level prediction was obtained for those isolates with countries represented in the training data (Ethiopia and India). For validation isolates that were sourced from countries in Central and South Asia (Afghanistan and Pakistan) and East Africa (Sudan, Uganda and Eritrea) that were not included in the SNP barcode, the predictions reflected the nearest countries included in the development process.

## Discussion

*Plasmodium vivax* accounts for a significant proportion of the global malaria burden, with the greatest incidence outside of sub-Saharan Africa [2]. The resilience of the parasite is evidenced by its re-appearance in regions where malaria transmission had previously been halted [9]. Microsatellite genotyping has been used to study *P*. *vivax* genomic and population dynamics, although it underestimates the total variability in natural populations [39]. Whole genome sequencing is the gold standard approach but is currently logistic- and cost-inefficient for large genomic epidemiological studies, especially in under-resourced endemic areas. We have a developed *in-silico* a 71 SNP barcode, informed by whole genome sequencing data from 433 isolates across 17 different countries worldwide. To date, barcodes in *Plasmodium* species have each been designed for specific roles. Some barcodes have focused on the determination of the geographical origin of the samples using nuclear or organellar genomic markers [29,32], others on measuring transmission intensity using the frequency of unique haplotypes [28,33] or the complexity of the infections [30]. Further, *P*. *vivax* barcodes have been based on small datasets featuring fewer geographic populations [34]. As more datasets become available genotyping tools such as our barcode will have global applicability. In fact, application of a 42-SNP barcode [33] to our dataset led to a lower accuracy (77.5%) when predicting origin of the isolates at a country level. The 42-SNP barcode also performed sub-optimally when estimating SNP-based relatedness of isolates, and therefore may not be suited to the inference of local transmission networks.

Our barcode was constructed by recognising that common SNPs are more robust markers and harbour greater explanatory power for both geographical and transmission inference, as demonstrated previously in human genetic studies [40]. By triaging SNPs using established LD tagging methods, it was possible to apply random forest methodology to select 60 markers, augmented by 11 inter-continental SNPs, to accurately predict country of source (91.4%). Whilst there are alternatives to the random forest algorithm, this approach has become an established data analysis tool in bioinformatics, with reported high performance in settings where the number of variables is much larger than the number of observations [38]. The methodology has an ability to explore complex interactions between correlated SNPs, and return useful measures of their predictive importance [41,42]. The set of “important” SNP predictors of country determined by the random forest approach was robust to initial model parameterisation, and the final model and set of SNPs were validated on a 20% subset of the original samples augmented by prospectively collected ones from Brazil. The 71 SNP barcode and intermediate SNP sets informing its construction were used to reconstruct principal component analysis plots. These plots were consistent with the overall population structure based on 720k SNP markers, and therefore confirm there was no major loss of geographical specificity.

Our 71 SNP barcode is the first that shows such strong levels of accuracy in geographic prediction at a country level in *P*. *vivax* for a diverse dataset, making it a valuable tool for the detection of imported cases of malaria. As whole genome data becomes available, especially from sites with currently poor coverage such as central America, Africa and South Asia, the machine learning approach can be used to update the SNPs in the barcode. The barcode was also designed to be able to provide information about potential transmission trends and therefore be useful in field settings, where large genomic differences are less likely, with the exception of imported cases. The proportion of unique haplotypes identified across the dataset was high (98.2%; 425/433 haplotypes observed), which allows greater scope for informing on intra-border haplotype diversity, including low diversity such as in an outbreak setting. A simulation study using *P*. *falciparum* genome sequencing and *P*. *vivax* microsatellite data has estimated that 200 biallelic markers should be used for IBD analysis [21]. In our study, using whole genome sequencing data on *P*. *vivax* we demonstrate the potential of a 71 SNP barcode to estimate genetic distance (SNP differences) as a measure of relatedness and to predict geographic origin to the country level. Further work could be aimed at assessing and expanding this set of markers and explore its potential use for IBD analysis specifically focusing on intra-border transmission. This potential utility for transmission characterisation was demonstrated by the use of intra-border highly related isolates, where we confirmed that the SNP distances based on the 71-SNP barcode and genome-wide 720k markers were highly correlated, and represent an improvement on comparative reported values for microsatellites (Pearson’s *r*^*2*^ = 0.70) [39]. Further, the use of the 71 SNP barcode was validated *in silico* using data from 60 *P*. *vivax* sourced from a low endemic and near-elimination setting in Malaysia, where it was possible to partition the population into highly structured subclades and confirm the presence of an outbreak cluster, previously identified using both microsatellite genotyping and sequencing. Also, by using the 71-SNP barcode we identified a misclassified “outbreak” isolate with an identical microsatellite haplotype, and evidence of regional clustering by the different administrative divisions, further supporting the utility of the tool and its superior performance to microsatellite genotyping. One of the challenges when studying *P*. *vivax* infections is the difficulty to differentiate recrudescent infections from reinfections or relapses [43–46]. Although, we have not explored the ability of our barcode to differentiate between such cases, based on the results from the Malaysian setting we anticipate it would at least be able to identify cases of relapse caused by meiotic siblings [44]. This is because of the high degree of similarity expected in such cases already picked up in clonal expansions, although specific work would be needed to address this challenge.

The barcode was also tested on 132 newly assessed isolates sourced from travelers to 10 different countries across the main *P*. *vivax* endemic regions, revealing that the 71 SNPs have a strong regional discriminatory power, and achieved an 80.1% overall accuracy in classifying the isolates at a country level for the countries represented in the barcode development. The PCA performed using the 71 SNPs showed that isolates originating from geographically distant regions (e.g. South Asia) clustered, and demonstrated the ability of the barcode to identify regional signatures even for previously unstudied locations. Nevertheless, the next step in order to improve our barcode would be the incorporation of the whole genome sequencing data from these isolates and future collections. Further, it is possible to genotype the barcoding SNPs using standard TaqMan genotyping assays or PCR followed by high resolution melting analysis, as previously described for other malaria SNP barcodes [27, 33]. It is also possible to apply amplicon sequencing using a portable sequencer, such as the MinION nanopore platform linked to a laptop computer. This approach was applied recently to genotype *P*. *falciparum* parasites [47].

In summary, we have presented a new *in-silico* molecular barcode for *P*. *vivax* that can provide information on both geographical origin and identify highly related isolates within country borders to help infer transmission events and identify foci of infection. The 71 SNP barcode out-competes previous genotyping methods and is a powerful and potentially affordable solution that could enable the execution of large genomic epidemiological studies, with high throughput assessment of large numbers of parasites. By leveraging off growing and large datasets of whole genome sequencing data, and the power of machine learning algorithms, it will be possible to update the barcode, augment it with drug resistance markers, and implement it rapidly in field-based settings using portable technologies. Ultimately, insights into genetic diversity will assist the much-needed understanding of the dynamics of *P*. *vivax* populations and inform disease control decision making.

## Materials and methods

### Genomic data generation

Illumina sequenced data from previously published studies [14–16] was downloaded from ENA repository to form a total dataset of 834 isolates. Some of these data are from the MalariaGEN *P*. *vivax* Genome Variation project. Each isolate data was mapped against the PvP01_v1 reference (obtained from http://genedb.org) using *bwa-mem* [47]. SNPs (n = 1,522,046) were called from the resulting alignments using the *samtools* software suite [48], as previously described [34]. Isolates and SNPs were excluded if they presented with > 20% missing or heterozygous genotype calls, and additional SNPs were removed if located within hypervariable gene regions (e.g. *vir* genes). The final dataset consisted of 720,340 high-quality SNPs and 433 isolates.

### Population structure and tag SNP selection

The 720,340 high-quality biallelic SNPs and its subsets were used to infer distance matrices by calculating Manhattan distances (”identity-by-state”) between samples. These genetic distances where divided by the sum of the allele frequencies of the SNPs included in their calculation, in order to make the units comparable. These matrices were used to generate principal component analysis (PCA) plots and neighbour-joining trees (R *ape* package [49]). The Pearson’s *r*^*2*^ metric, calculated using the R base function *cor*, was used to estimate the correlation between distance matrices. A Bland-Altman analysis comparing genetic distances based on different sets of SNPs was performed using the R *BlandAltmanLeh* library. The software *TAGster* [37] was used to identify SNPs which summarise blocks of high linkage disequilibrium, as estimated using the genetic *r*^*2*^ metric. We specified an *r*^*2*^ threshold of at least 0.7 for inclusion in a block and a window size of 500 kbp, leading to 16,110 SNPs with minor allele frequency > 0.3 being included for analysis. The resulting 1,173 tag SNPs identified were then further characterized for downstream analysis. The Fixation index (*F*_*ST*_) was calculated using in house R scripts and a threshold of *F*_*ST*_ > 0.7 was used to determine population-level informative barcoding SNPs [25,34]. A flow diagram summarising the selection process of the SNPs is presented in S7 Fig.

### Barcode SNP selection using a random forest approach

The selected 1,173 highly informative SNPs obtained using a combination of minor allele frequency and SNP tagging approaches were extracted from the original dataset. Allele imputation was performed on the missing data points (4.2%) in the dataset using the R *missForest* package [50]. This yielded an estimated out-of-bag error in the imputation of 16.2% which left a total of 0.7% potentially erroneous calls. After imputation, a random selection of 80% of the dataset was assigned as a training set and the remaining 20% was assigned as a test dataset. Source country was tested as the predicted variable. Subsequently, five hundred trees were calculated using the *RandomForest* [51] package in R in order to determine the SNPs in the dataset with highest importance for classification of samples into countries. We then selected the 60 SNPs with highest importance and used the R *LDcorSV* package [52] to calculate the correlation between the markers. PCA plots and neighbour-joining trees were constructed for this subset of SNPs. The final subset of 71 SNPs was then used to retrain the random forest model on the training set, and this model was applied for the prediction of the country of origin in the validation dataset.

### Validation of the barcode using UK traveller isolate genotype data

DNA was extracted from blood samples sourced from 132 returning travellers to the UK from endemic areas between 2017 and 2018. The 132 samples were sourced from travellers to Afghanistan (n = 26), Bangladesh (n = 1), Eritrea (n = 11), Ethiopia (n = 6), Guyana (n = 3), India (n = 38), Pakistan (n = 35), the Philippines (n = 1), Sudan (n = 7) and Uganda (n = 4). The blood samples are stored in the Public Health England (PHE) Malaria Reference Laboratory (MRL) at the LSHTM. The DNA underwent Illumina MiSeq 150bp paired-end sequencing at the LSHTM. The resulting data was aligned to the *P*. *vivax* PvP01_v1 reference genome using *bwa-mem* (see [34,53] for the bioinformatics pipeline), thereby allowing the calling of the genotypes at the 71 positions (S2 Table). The validation isolates were not used in the construction of the random forest model. The PHE and LSHTM ethics boards provided approval for the sequencing of the *P*. *vivax* DNA.

## Supporting information

S1 Table The 433 high-quality *P*. *vivax* isolates used for the development of the barcode. (XLSX)

S2 Table The barcoding genotypes and country metadata for the 132 *P*. *vivax* prospective UK traveller isolates. (XLSX)

S1 Fig (Left) Distribution of SNPs according to minor allele frequency (MAF); (Right) SNPs partitioned into three equally sized divisions based on MAF (blue dashed lines), and a cut-off of MAF > 0.3 (red dashed line) was used to pre-select SNPs for downstream analysis. (TIFF)

S2 Fig (Top) Neighbour-joining tree based on 1,173 tagging SNPs in *P*. *vivax* selected using the *TAGster* [37] software shows strong similarity with the tree from Fig 2 (right), observing a strong geographical signal; (Middle) the correlation of genome distance based on whole genome sequencing (WGS; 720k SNPs) with the subset of tagging (1,173) SNPs is high (Pearson’s *r*^*2*^ = 0.98); (Bottom) A Bland-Altman analysis that compares the differences in genetic distance between those based on the whole genome and the subset of 1,173 tagging SNPs; it shows a slight underestimation of the distance measured by the tagging SNPs (mean of differences: 0.177; with standard deviation (SD)). (TIFF)

S3 Fig (A) Classification error for the different geographic categories across the 500 trees in the random forest model reaches stability when 100 trees are averaged; (B) Variable importance estimated from the random forest model for the number of 1,173 tagging SNPs. The red dashed line is the cut-off based on importance, which is the threshold used to determine SNP inclusion in the barcode.
(TIFF)

S4 Fig The low linkage disequilibrium (LD) between the 71 SNPs in the *Plasmodium vivax*. An overall low correlation (LD) was found between the 71 SNPs (mean linkage *r*^*2*^ = 0.15). LD blocks were observed and correspond to SNPs with geographic signal (i.e. Southeast Asian high frequency SNPs).
(TIFF)

S5 Fig The principal component analysis (PCA) plot and neighbour-joining tree constructed using a previously published 42-SNP barcode [33]. It shows ambiguous geographic clustering of *P*. *vivax* isolates. Geographical clustering by region was apparent, although a degree of overlap was observed and separation by country was not clear. This result is suggested by the low accuracy (77.5%) obtained when predicting geographical origin using a random forest model formed with the set of 42-SNPs.
(TIFF)

S6 Fig The principal component analysis (PCA) plot for the 132 *P*. *vivax* prospective UK traveller isolates, constructed using the 71 barcoding SNP genotypes. There is clustering by geographical region, including between Eastern Africa countries (Ethiopia, Eritrea, Sudan and Uganda), South/Central Asia (Pakistan, India, Bangladesh, Afghanistan), Guyana (South America) and the Philippines.
(TIFF)

S7 Fig Flow diagram of the SNP selection process for the 71 SNP barcode. (TIFF)
