# Supplementary figures and images for: A molecular barcode to inform the geographical origin and transmission dynamics of Plasmodium vivax malaria
Source: PLoS Genet. 2020 Feb 13;16(2):e1008576. doi: 10.1371/journal.pgen.1008576 (PMC7043780; doi:10.1371/journal.pgen.1008576)

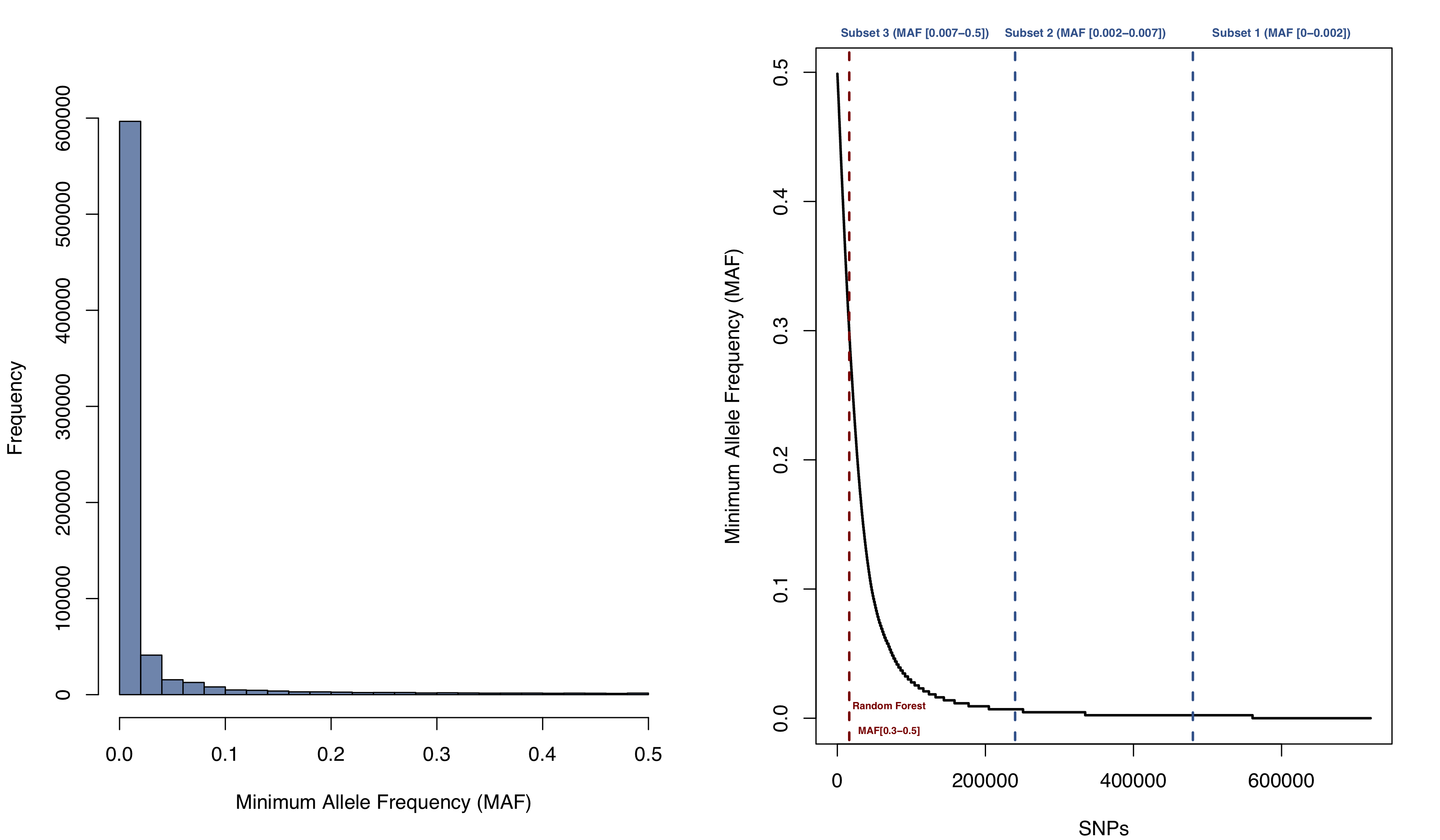

Supplement: S1 Fig — (TIFF) [file pgen.1008576.s003.tiff]

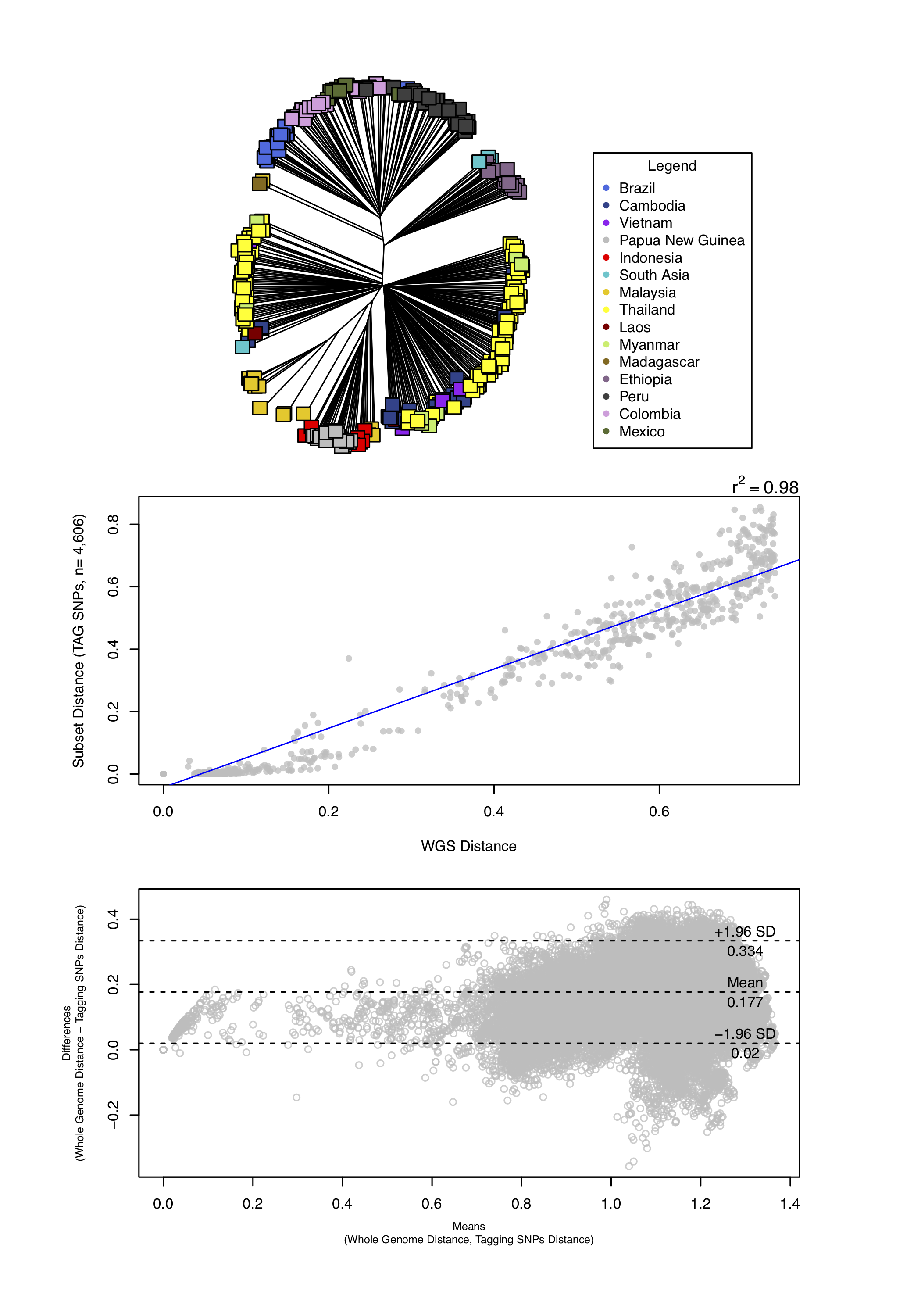

Supplement: S2 Fig — (TIFF) [file pgen.1008576.s004.tiff]

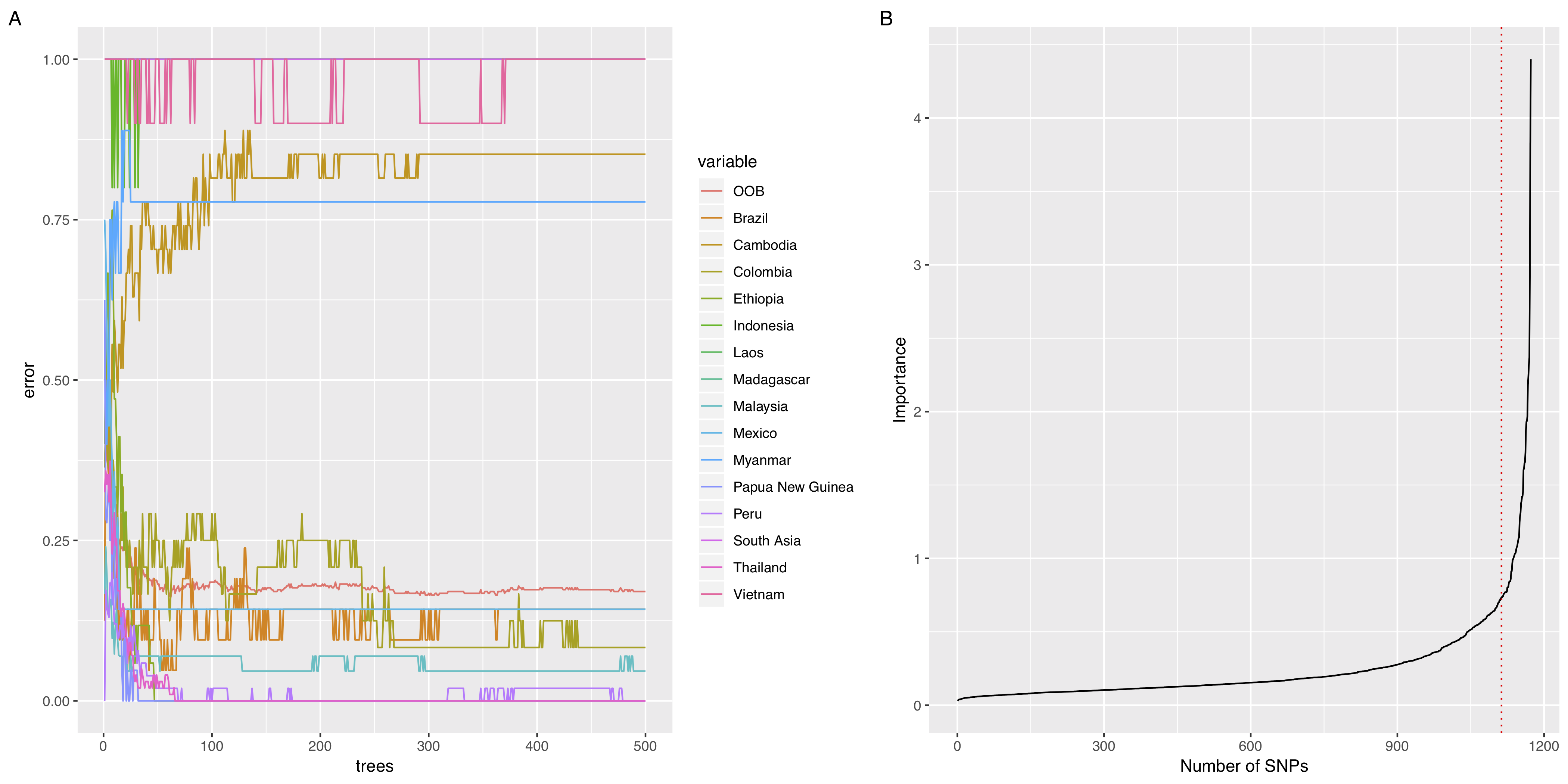

Supplement: S3 Fig — The red dashed line is the cut-off based on importance, which is the threshold used to determine SNP inclusion in the barcode. (TIFF) [file pgen.1008576.s005.tiff]

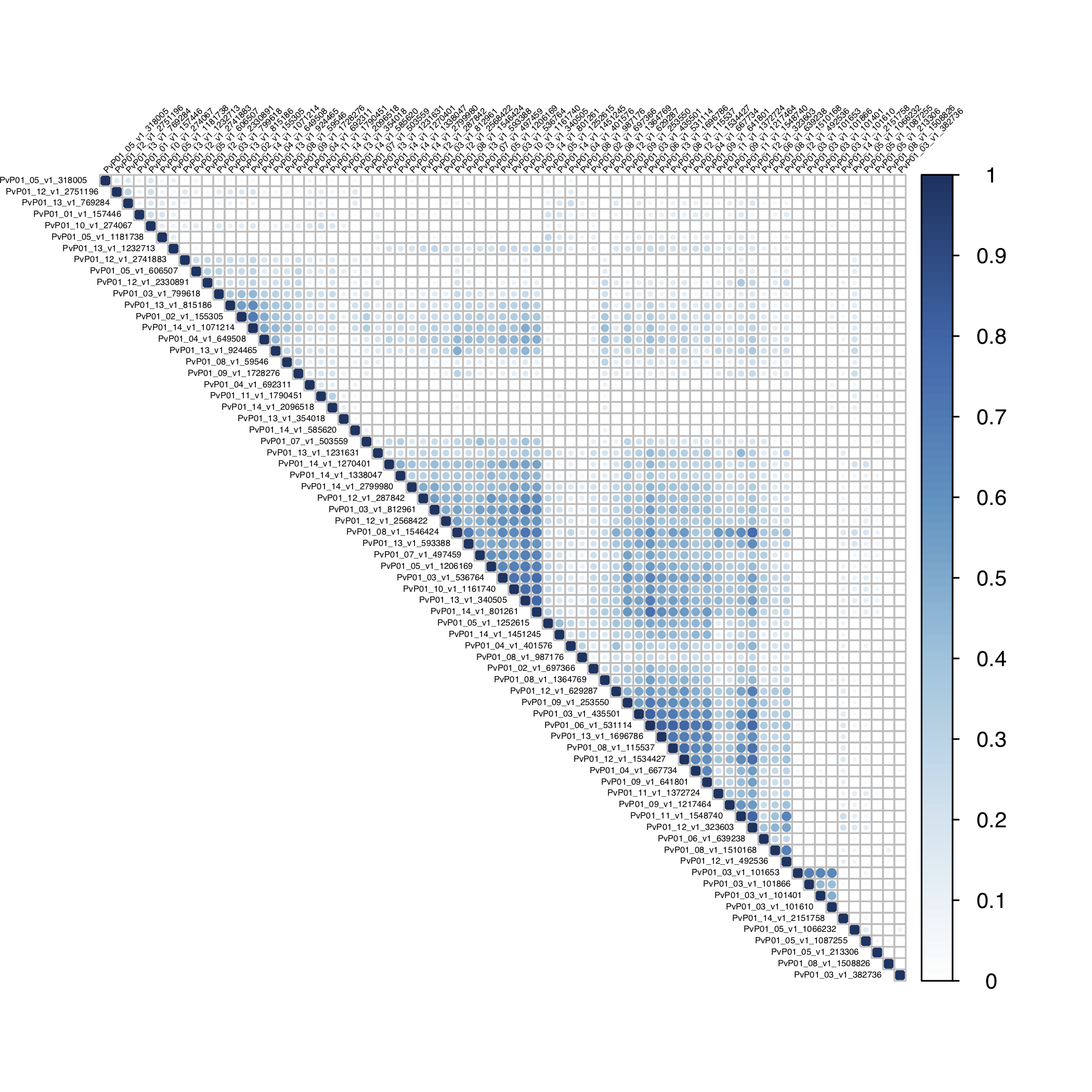

Supplement: S4 Fig — An overall low correlation (LD) was found between the 71 SNPs (mean linkage r2 = 0.15). LD blocks were observed and correspond to SNPs with geographic signal (i.e. Southeast Asian high frequency SNPs). (TIFF) [file pgen.1008576.s006.tiff]

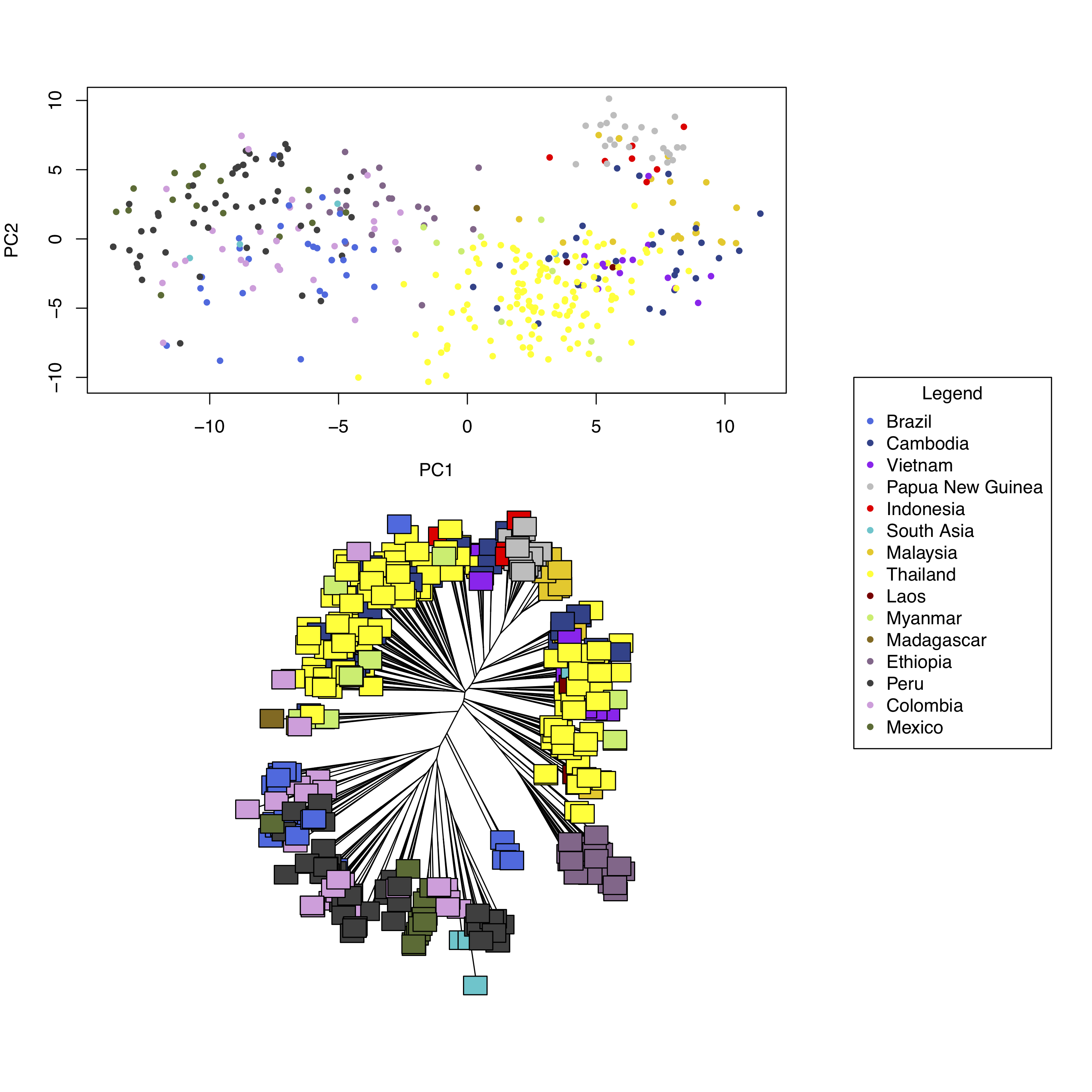

Supplement: S5 Fig — It shows ambiguous geographic clustering of P. vivax isolates. Geographical clustering by region was apparent, although a degree of overlap was observed and separation by country was not clear. This result is suggested by the low accuracy (77.5%) obtained when predicting geographical origin using a random forest model formed with the set of 42-SNPs. (TIFF) [file pgen.1008576.s007.tiff]

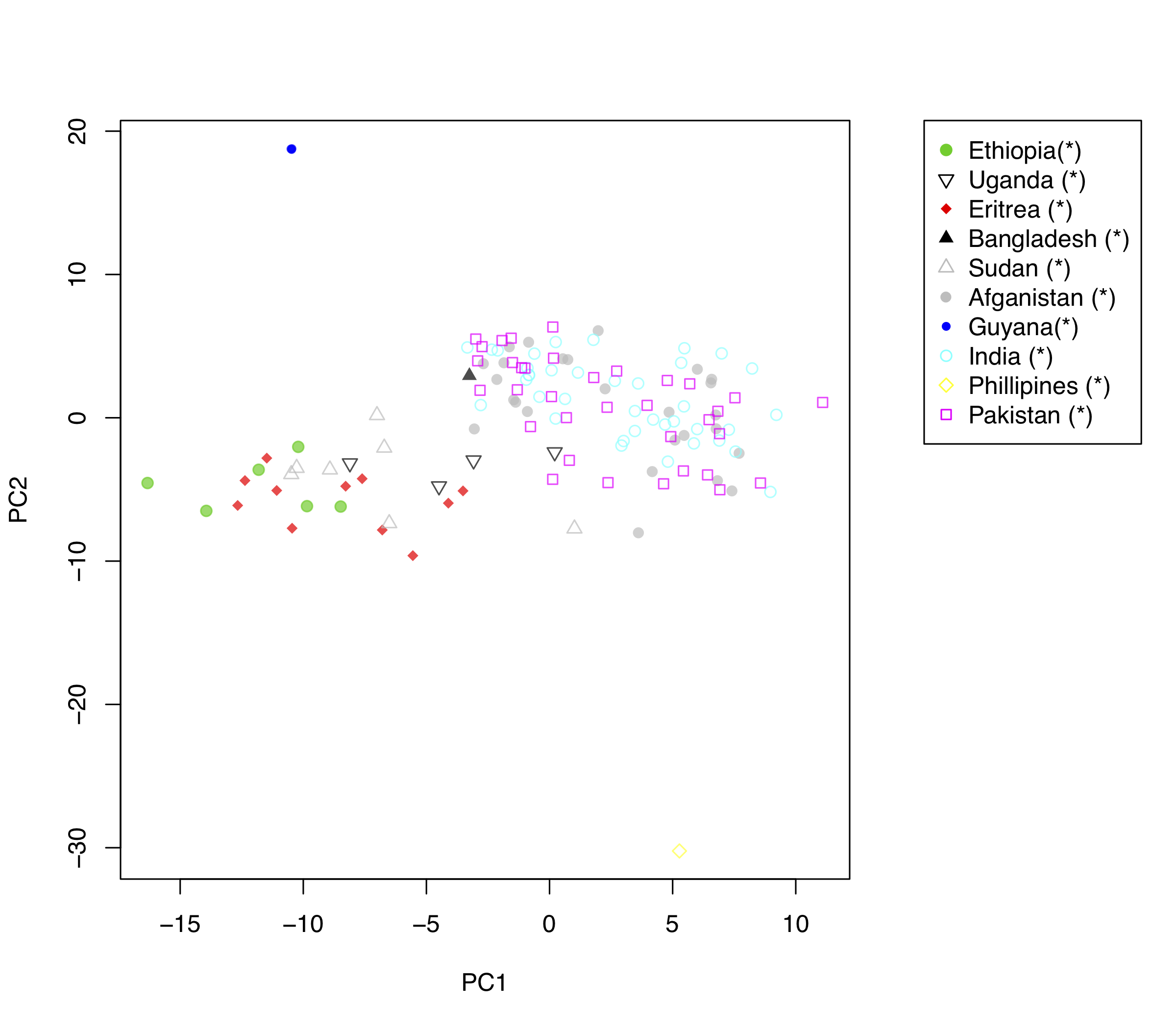

Supplement: S6 Fig — There is clustering by geographical region, including between Eastern Africa countries (Ethiopia, Eritrea, Sudan and Uganda), South/Central Asia (Pakistan, India, Bangladesh, Afghanistan), Guyana (South America) and the Philippines. (TIFF) [file pgen.1008576.s008.tiff]

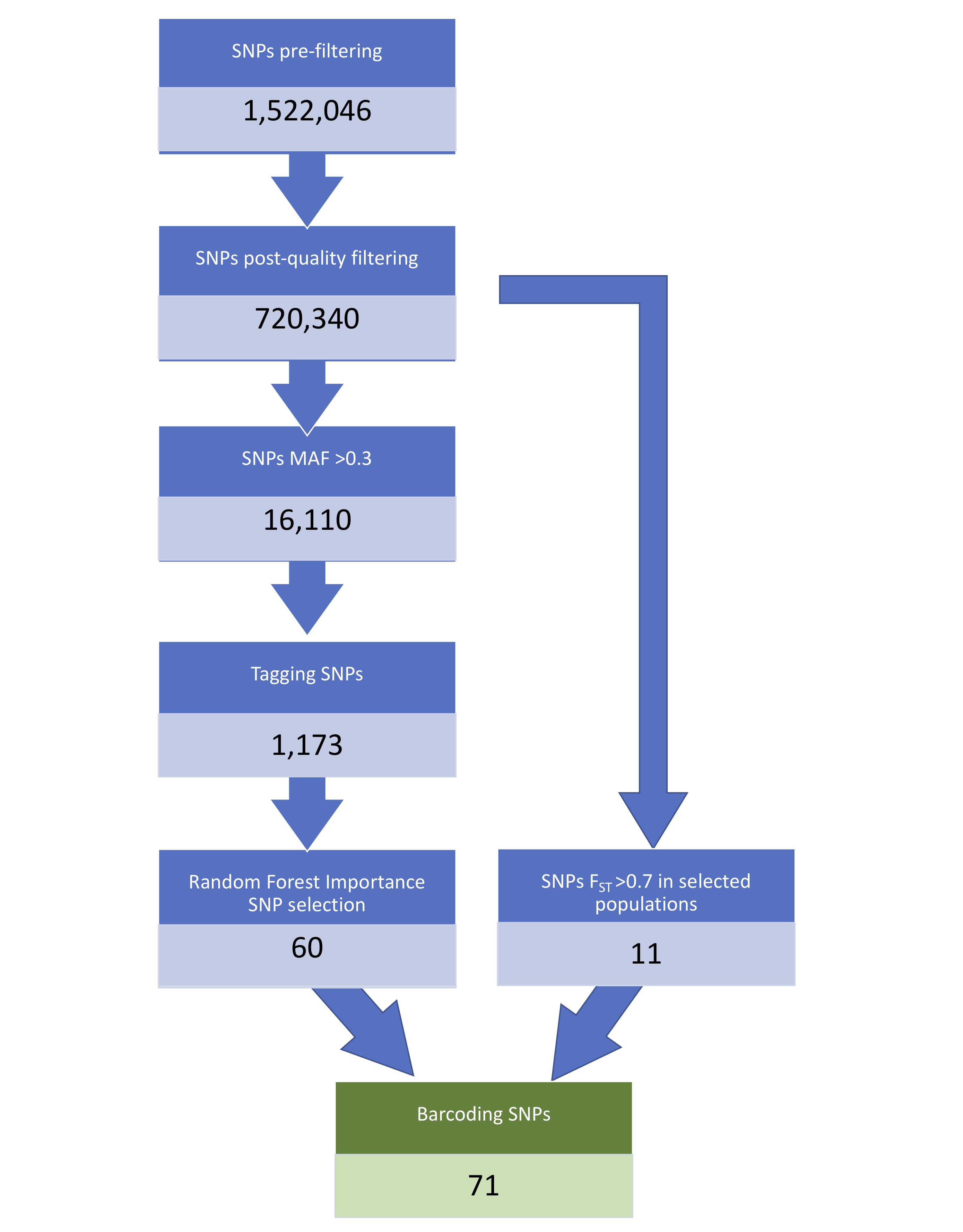

Supplement: S7 Fig — (TIFF) [file pgen.1008576.s009.tiff]
